# Predictive Strength of Contextual and Personal Variables in Soccer Players’ Goal Orientations

**DOI:** 10.3390/ijerph18179401

**Published:** 2021-09-06

**Authors:** Enrique Iglesias-Martínez, Jorge Roces-García, David Méndez-Alonso

**Affiliations:** 1 Faculty of Teacher Training and Education, Facultad Padre Ossó, University of Oviedo, 33003 Oviedo, Spain; iglesiasenrique@uniovi.es (E.I.-M.); mendezdavid@uniovi.es (D.M.-A.); 2 Polytechnic School of Engineering of Gijón, University of Oviedo, 33204 Gijón, Spain

**Keywords:** motivation, performance, team sport, psychology, self

## Abstract

Psychological variables, such as perceived motivational climate, goal orientation, self-determined motivation, and personality, have an influence on sports success performance. This study aimed to examine the relationships among a set of psychological variables (perceived motivational climate, goal orientation, self-determined motivation, and personality) in male and female footballers. Participants were 167 footballers (106 male, 61 female), aged 12 to 26, competing with clubs in the Spanish Football League. They all took four questionnaires aimed at evaluating motivational climate, goal orientations, self-determined motivation, and personality. The analyses of correlation and regression showed statistically significant relations among the variables. Neuroticism and psychoticism negatively relate to mastery motivational climate, the best predictor of self-determined motivation. It was concluded that contextual variables carry more weight in predicting goal orientations and self-determined motivation among participant footballers.

## 1. Introduction

Psychology has developed and consolidated its presence in the spectrum of the practice of competitive, professional, and amateur sports, and with it, the growth in the analysis of psychological variables in the sports context [1,2]. Within the sports context, motivation and its relation with sports performance have attracted the interest of numerous researchers [3], specifically with respect to the reasons individuals pursue certain goals [4]. For decades, the importance of this variable has been highlighted in the self-determination theory (SDT) of motivation [5,6], in which an explanatory model is postulated and widely used in sports psychology to provide answers regarding involvement in the performance of activities. SDT analyses the extent to which people engage with their actions through a high level of reflection and self-determined motivation. It establishes a continuum between demotivation or absolute lack of motivation and self-determined motivation [7]. The concept of self-determination has been widely used to explain sports behaviour [8]. This variable is measured through the self-determination index (SDI), and implies that high SDI scores have positive effects on the training practices of athletes [9].

The motivational processes that operate in the behaviours of athletes in different contexts have been explained mainly through SDT, but also through achievement goal theory [10,11]. Studies on achievement goal theory [12] have pointed to the importance of the motivational climate over self-determined motivation and development of positive behaviours. ‘Motivational climate’ [13] is a term that describes the environment created by the trainer, family, and environment in the sports context, distinguishing between perceptions of execution and the mastery climate. The motivational performance climate promotes social comparison as the basis for success, and the rewards are based on the demonstration of superior performance [14]. In the mastery climate, trainers promote satisfaction, interest, and intrinsic motivation [14] or self-determined motivation [15].

Similarly, achievement goal theory [12] relates the perceived motivational climate to athletes’ goal orientation. At least two goal orientations are assumed to exist, namely, a task (or mastery) orientation, in which competence is defined in terms of self-referenced criteria, and an ego orientation, which defines competence using external comparison criteria. The relevance of these goal orientations is demonstrated in sport, associated with different cognitions and behaviours, in which improving, learning, or mastering a task is the goal of task-oriented people, as opposed to ego-oriented ones, who pursue social approval and perform external tasks for results [16].

In the present study, we aimed to incorporate the importance of personality in the sports field, as great effects have been observed between personality, success, and sports progression [17,18]. For example, personality profiling in sport has been shown to be beneficial with established athletes [19] and, in turn, facilitates the transition of individuals from youth to adult teams [20]. To study how this variable acts as a modulator of goal orientation and to observe its relation with motivational climate and self-determined motivation, we used the three-dimensional personality model of Eysenck [21], in which two personality dimensions can be distinguished—extroversion–introversion and neuroticism–stability. A third dimension with independent functionality, called psychoticism, is also covered. This model is of a psychobiological rather than contextual character; thus, a priori, its study seemed interesting in relation to contextual variables within the sports environment. According to this model, an individual with high scores in neuroticism refers to an anxious, emotional person with a tendency to worry, whereas low scores characterize emotionally stable people, who easily identify their emotions, are responsible, and have flexibility to change. Along these same lines, a high score in extroversion is associated with greater sociability, little tendency to remain alone, and preference for strong emotions and optimism, whereas low scores imply withdrawal and inability in social skills. Finally, low scores in the psychotic dimension determine a tendency for dependence and the inability to act in decision making, in contrast to high scores, which characterize insensitive, inhuman, antisocial, violent, aggressive, and extravagant people [21]. These general characteristics create a psychological system produced by the interaction and subsequent adaptation between the individual and the environment [22]. In sports, such an interactive process takes place in the same way, where personality development is generated by the interaction between an individual’s genetics and the environmental influence of physical activity [23], producing a bidirectional association between an athlete’s personality and sports practice [24]. According to this bidirectional hypothesis [23,24], personality traits are influenced by the practice of sport (i.e., by contextual variables), as the athlete’s adaptation to his or her sporting environment becomes important.

Within this frame of reference, the objectives of this study were to analyse the relationships among perceived motivational climate, self-determined motivation, goal orientations, and personality in a collective sport, such as soccer, to determine whether these variables have a predictive weight vis-à-vis the others and to examine differences in the aforementioned variables by gender (men and women) and category of sports.

## 2. Materials and Methods

### 2.1. Participants

A total of 167 footballers (106 males and 61 females), between 12 and 26 years old (M = 15.53 years, s.d. = 2.78 years), who belonged a to soccer team (47 children’s category (12–13 years old), 41 cadet (14–15 years old), 28 youth (16–18 years old), 47 professional categories (>18 years old)) participated in this research.

### 2.2. Instruments

The information was gathered using five types of specialised and validated surveys (PMCSQ-2, POSQ, PLOC, Junior, EPQ-J and Adult, EPQ-A):

- Perceived Motivational Climate in Sports Questionnaire-2 (PMCSQ-2). To measure perceptions of motivational climate, the Spanish-adapted version of PMCSQ-2 Newton [25] was used. The adapted version, constituted by two factors, called mastery and execution [26], consists of 33 items: 17 items for mastery climate (e.g., the main thing is to improve) and 16 items on execution climate (e.g., the trainer only looks at the best), with a Cronbach’s alpha value of 0.84. The response mode is Likert type, with scores ranging from 1 (strongly disagree) to 5 (strongly agree). In the present study, Cronbach’s alpha values of 0.84 for the mastery climate and 0.87 for the execution climate were obtained.
- Perception of Success Questionnaire (POSQ) [27]. This scale consists of 12 items: six goal-to-task orientation items (e.g., demonstrates clear personal improvement), and six ego or result orientation items (e.g., I am clearly better than the others). The questionnaire is answered on a Likert scale, with scores ranging from 1 (totally disagree) to 5 (totally agree). In the present study, Cronbach’s alpha values of 0.76 for task orientation and 0.85 for ego orientation were obtained.
- Perceived Locus of Causality Scale (PLOC) [28]. The adapted version consists of 20 items (four per factor) that measure intrinsic motivation, identified regulation, introjection, external motivation, and demotivation [29]. The scale is headed by the statement “I participate in the training sessions...” and is answered through a Likert-type scale from 1 (totally disagree) to 7 (totally agree). The scores obtained were used to calculate the SDI: [(2 × intrinsic motivation + identified regulation) − [(introjected regulation + external regulation)/2 + 2 × demotivation)] [30]. In the present study, the SDI yielded a Cronbach’s alpha value of 0.79.
- Eysenck Personality Questionnaire (Junior, EPQ-J and Adult, EPQ-A) [21]. This is a questionnaire of individual or collective application, aimed at respondents aged 8 to 15 years (EPQ-J) and 16 years and older (EPQ-A). Its purpose is to evaluate the three personality dimensions of neuroticism, extroversion, and psychoticism. The youth version has 81 items and the adult version has 92. In both versions, the answer options are dichotomous (yes/no). In the present study, Cronbach’s alpha values in EPQ-A of 0.81 for neuroticism, 0.67 for extraversion, and 0.70 for psychoticism were obtained. For EPQ-J, values of 0.78 for neuroticism, 0.65 for extraversion, and 0.70 for psychoticism were obtained.

### 2.3. Procedure

Athletes completed the questionnaires, following the same instructions, at the facilities provided by the soccer clubs. Collaboration was requested; we asked them for sincerity, reflection, and attention to each of the questions. Informed consent was obtained from the parents or guardians of underage athletes prior to completion of the questionnaires applied in the study. Participation was voluntary and all information was treated confidentially and anonymously. The application was carried out individually or in a small group, always face-to-face, depending on the availability of the athlete and the club, and always under the supervision of the authors of the study.

### 2.4. Data Analysis

The data were analysed using SPSS 25.00 (IBM, Armonk, NY, USA). Descriptive analyses and bivariate correlations were performed for each of the eight variables: neuroticism, extroversion, psychoticism, mastery climate, execution climate, ego orientation, task orientation, and SDI. To analyse differences by gender and category, multivariate analyses of variance were carried out (taking gender as an independent variable in the first position and the sports category in the second position). Finally, to study the extent to which personality variables and perceived motivational climate predict goal orientations and self-determined motivation, a hierarchical regression analysis was performed using the stepwise method.

## 3. Results

### 3.1. Descriptive Statistics and Bivariate Correlations

The means and standard deviations for all variables are presented in Table 1.

After analysis, the following relations were found to be statistically significant. Neuroticism was positively related to psychoticism, and both were negatively related to extroversion. Psychoticism and neuroticism were positively related to the execution climate and goal orientation towards the ego; they were negatively related to mastery climate and SDI. Mastery climate was negatively related to execution climate and goal orientation to the ego, and positively to goal orientation to the task and SDI. For its part, execution climate was positively related to goal orientation to self and negatively related to SDI.

### 3.2. Gender and Category Differences

When a 2 (gender: 1 = male, 2 = female) × 5 (category: 1 = children, 2 = cadet, 3 = juvenile, 4 = senior 5 = 1st Division) multivariate analysis of variance was performed, a significant multivariate effect emerged for gender, λ_Wilks_ = 0.872, *F*(8,124) = 2.27, *p* < 0.05, *η*^2^ = 0.13. Meanwhile, univariate analyses showed differences in the mastery climate, *F*(1,140) = 5.38, *p* < 0.05, *η*^2^ = 0.04, where females scored higher than males. Regarding the execution climate, *F*(1,140) = 5.33, *p* < 0.05, *η*^2^ = 0.04, and ego orientation, F(1,140) = 9.23, *p* < 0.05, *η*^2^ = 0.07, males scored higher than females. A significant multivariate effect also emerged for the category λ_Wilks_ = 0.655, *F*(32,458) = 1.74, *p* < 0.01, *η*^2^ = 0.10.

Univariate analyses showed differences in extroversion, *F*(1,140) = 8.86, *p* < 0.001, *η*^2^ = 0.21; that is to say, as the category in which they play progressed (from children to professional), the score on this variable decreased. Finally, no significant differences were observed in gender interaction by category λ_Wilks_ = 0.799, *F*(24,360) = 1.74, *p* = 0.23, *η*^2^ = 0.07.

### 3.3. Hierarchical Regression Analysis

To determine whether the predictive value of personal goal orientation is greater depending on personal or contextual characteristics, a regression analysis was carried out in successive steps (Table 2). Personality variables were introduced, followed by motivational climate variables. SDI was also included as a dependent variable. Of the three dependent variables analysed, only neuroticism predicted ego orientation in step 1; it then disappeared in step 2. The most important predictor of this variable was the execution climate; whereas for task orientation and SDI, the most important predictor was the mastery climate. Thus, contextual variables were the ones that could predict these dependent variables.

## 4. Discussion

Derived from the study of correlation between the study variables, statistically significant relations were found. Within the framework of the theory of motivational climate achievement goals [25], the dimensions of climate, mastery climate (involvement in the task), and execution climate (ego-centred) were included. The results indicated that both types of climate correlated negatively at the opposite end of a continuum. However, given the negative correlation between mastery climate and execution climate, it is appropriate, in a collective sport such as soccer, to enhance the climate of mastery, especially because the presence of a climate of execution would imply the predominance of individual objectives focused solely on the self [31]. The results of the relation between motivational climate and goal orientations indicated that mastery climate was positively related to goal orientation to the task and negatively related to goal orientation to the ego [32]. Thus, the socialization or climate generated by the trainer and the environment has important implications for the way to interpret success and define competence (strengthening goals directed towards the task when the climate favours it). In contrast, execution climate did not show a significant relation with goal orientation to the task. The explanation could be related to personal predisposition (centred on the self or ego), which, to a great extent, would determine the a priori probability of adopting a concrete goal and of representing a pattern of behaviour [33]. These relations confirm the need to promote a motivational climate of mastery in a team sport such as soccer, which will be related to goal orientations to the task more than to individual ones. Therefore, the facets related to the type of goal orientation would be those included in the most adaptive social pattern. When the practice of sports, for example, becomes professional or competitive, a progressive increase in the execution climate would be observed owing to greater rivalry associated with extrinsic goals of greater strength [11].

The results of the correlation analysis showed significant relations between the motivation and variables such as personality dimensions and SDI. Specifically, the mastery climate showed a statistically significant positive relation with SDI (as opposed to the execution climate, whose statistical relation was negative). It should be noted that high SDI scores have numerous positive implications for athletes’ training practices [34,35]. Likewise, we found that psychoticism and neuroticism were positively related to the execution climate and goal orientation towards the ego, whereas both negatively predicted a mastery climate [36]. All these data, as a whole, showed that these two personality variables negatively affected both self-determined motivation and the climate of mastery, and consequently, task orientation. This tendency may hinder sporting success; variables such as neuroticism are lower in professional athletes [37].

Regarding personality variables, neuroticism was significantly and positively related to psychoticism, and both showed a significant and negative association with extroversion. These results confirmed those in similar studies, in which athletes scored high in extroversion and low in neuroticism, leading to the negative relation between these variables [38]. One aspect to comment on is the relative proximity between the scales of neuroticism and psychoticism. This proximity could be explained by considering that the aspects shared by both dimensions are closer to the extroversion dimension [39]. Likewise, psychoticism and neuroticism correlated positively with execution climate and ego orientation, and negatively with mastery climate and SDI. In the same way, SDI had a positive relation with task orientation and a negative relation with ego orientation. Therefore, these two personality variables would negatively affect the self-determined motivation, climate of mastery, and task orientation.

The results of the regression analysis supported the findings described above. Thus, neuroticism initially predicted an ego-centred orientation, whereas mastery climate predicted a task-centred orientation. Furthermore, neither neuroticism nor psychoticism showed a predictive power over SDI, but mastery climate did predict this variable (SDI). It should be pointed out that self-determined motivation is also relevant outside the sports field, given that it is related to a reduction in depressive symptoms [40].

As previously stated, contextual and dispositional variables in the sports context are related in the so-called bidirectional hypothesis [23]. According to this hypothesis, certain personality traits increase with the regular practice of sport, but sports performance also contributes to personality enrichment [24]. To delve more deeply into this two-way influence, regression analysis was used to analyse whether contextual variables predicted personal variables, or if the opposite occurred. The results indicated that both mastery climate and execution climate could predict SDI (with a greater significance in the case of mastery climate), thereby leading to the conclusion that the contextual variables had greater weight in the relationship. Based on these results, it is clear that the coach can have a positive impact on the intrinsic motivation and personality dimensions, and as such, the value of this variable must be emphasised.

Furthermore, gender and category differences were analysed. With respect to gender, a general tendency was observed in women, who obtained higher scores in the mastery climate than men. Meanwhile, the men scored higher in the execution climate and ego orientation. The differences obtained can be explained by social factors. Women tend to interpret sports in a cooperative and leisure context, as opposed to men, for whom the competitive aspect in sports seems predominant [41]. With respect to this last dimension of personality, extroversion decreased in both genders as the category in which they played progressed. From this, it is inferred that the longer they practice sports, the less extroversion they would show. This relation differs from findings in earlier studies: sports practice is associated with extroversion [42], athletes score high on extroversion [38,43], and athletes in collective sports are more extroverted [37]. In any case, influences on sports participation are well established [44], and personality interacts with environmental changes, affecting participation in sport and physical activity. For this reason, it is necessary to emphasise that the present participants were footballers of diverse categories. All belonged to professional clubs of the Spanish soccer league, in which, beginning in the initial categories, the footballers not only compete with external teams but also with members of their own team or companions (to enjoy more minutes, more matches, promotion, etc.). This context influences the different dispositional variables of the sportsman [31], as happens with extroversion, as the player advances to higher categories.

Finally, it is important to point out certain limitations of the present study, such as sample size, which, in some of the categories, was small. A greater number of participants would make it possible to specify the differences according to degree or professional level. Moreover, no measure of performance was included. It ought to be noted that participants of many different ages were surveyed for this study. Not all of them played at a professional level, which might influence our interpretation of the results. Thus, a future line of interest is to deepen the relationships between the variables studied here through longitudinal studies, which could further explore influential or predictor variables of sporting success, such as psychological constructs or sociodemographic, familiar, or academic variables.

## 5. Conclusions

The team environmental and leadership variables will configure a type of goal orientation and affect additional personality factors. Based on the results of this study, it seems obvious that the coach has a positive influence on intrinsic motivation and on the personality dimensions. It is, therefore, necessary to emphasize the value of this variable. This may result in a series of consequences, which play an important role as transmitters of values, attitudes, and behaviours and could have an impact on sports performance. Data found in this study could be used to create new psychological interventions with aims to enhance soccer development.

## Figures and Tables

**Table 1:** Descriptive analyses and bivariate correlations.

	M	DT	1	2	3	4	5	6	7
1. Neuroticism	9.11	4.79	1						
2. Extroversion	17.37	4.12	−0.32 **	1					
3. Psychoticism	3.86	3.18	0.45 **	−0.19 *	1				
4. Mastery climate	4.30	0.49	−0.26 **	0.17 *	−0.16 *	1			
5. Execution climate	2.43	0.67	0.32 **	−0.15	0.18 *	−0.52 **	1		
6. Ego orientation	3.26	1.00	0.21 **	−0.06	0.21 **	−0.26 **	0.40 **	1	
7. Task orientation	4.60	0.47	−0.05	0.04	0.02	0.29 **	0.02	0.22 **	1
8. SDI	8.41	2.40	−0.17 *	0.14	−0.21 **	0.50 **	−0.36 **	−0.16 *	0.35 **

Legend: M = mean, s.d. = standard deviation. Note: * *p* < 0.05, ** *p* < 0.001.

**Table 2:** Hierarchical regression analysis (dependent variables: target orientations; independent variables: personality variables, dimensions of motivational climate, and SDI).

	Ego			Task		SDI	
	Variables	Β	R^2^	Β	R^2^	Β	R^2^
Step 1	Neuroticism	0.040 *	0.032	−0.096	0.013	−0.069	0.044
	Extroversion	−0.030		0.050		0.108	
	Psychoticism	0.034		0.089		−0.104	
Step 2	Neuroticism	0.032	0.174	−0.088	0.154	0.059	0.274
	Extroversion	−0.002		0.029		0.075	
	Psychoticism	0.027		0.099		−0.081	
	Mastery climate	−0.125		0.438 ***		0.390 ***	
	Execution climate	0.530 ***		0.270 **		−0.177 *	

Note: * *p* < 0.05, ** *p* < 0.01, *** *p* < 0.001.

## Data Availability

The data presented in this study are available on request from the corresponding author.

## References

[1] Benítez-Sillero J., Martínez-Aranda L.M., Sanz-Matesanz M., Domínguez-Escribano M. (2021). Determining Factors of Psychological Performance and Differences among Age Categories in Youth Football Players. Sustainability.

[2] Han D.H., Kim S.M., Zaichkowsky L. (2013). Insecure attachment and anxiety in student athletes. J. Sports Med. Phys. Fitness.

[3] Clancy R.B., Herring M.P., MacIntyre T.E., Campbell M.J. (2016). A review of competitive sport motivation research. Psychol. Sport Exerc..

[4] Gaudreau P., Braaten A. (2016). Achievement Goals and their Underlying Goal Motivation: Does it Matter Why Sport Participants Pursue their Goals?. Psychol. Belg..

[5] Deci E.L., Ryan R.M. (2000). The “What” and “Why” of Goal Pursuits: Human Needs and the Self-Determination of Behavior. Psychol. Inq..

[6] Ryan R.M., Deci E.L. (2017). Self-Determination Theory: Basic Psychological Needs in Motivation, Development, and Wellness.

[7] Pelletier L.G., Tuson K.M., Fortier M.S., Vallerand R.J., Brikre N.M., Blais M.R. (1995). Toward a New Measure of Intrinsic Motivation, Extrinsic Motivation, and Amotivation in Sports: The Sport Motivation Scale (SMS). J. Sport Exerc. Psychol..

[8] Hagger M.S., Chatzisarantis N.L.D. (2007). Advances in self-determination theory research in sport and exercise. Psychol. Sport Exerc..

[9] Irwin B.C., Feltz D.L. (2016). Motivation gains in sport and exercise groups. Routledge International Handbook of Sport Psychology.

[10] Atkins M.R., Johnson D.M., Force E.C., Petrie T.A. (2015). Peers, parents, and coaches, oh my! The relation of the motivational climate to boys’ intention to continue in sport. Psychol. Sport Exerc..

[11] Jaakkola T., Ntoumanis N., Liukkonen J. (2016). Motivational climate, goal orientation, perceived sport ability, and enjoyment within Finnish junior ice hockey players. Scand. J. Med. Sci. Sports.

[12] Nicholls J.G., Roberts G.C. (1992). The general and the specific in the development and expression of achievement motivation. Motivation in Sport and Exercise.

[13] Ames C. (1992). Classrooms: Goals, structures, and student motivation. J. Educ. Psychol..

[14] Theodosiou A., Mantis K., Papaioannou A. (2008). Student self-reports of metacognitive activity in physical education classes. Age-group differences and the effect of goal orientations and perceived motivational climate. Educ. Res. Rev..

[15] Cecchini J.A., Mendez-Gimenez A., Fernandez-Rio J. (2014). Effects of Epstein’s TARGET on adolescents’ intentions to be physically active and leisure-time physical activity. Health Educ. Res..

[16] Modroño C., Guillen F. (2016). Motivation and self-concept in windsurfers: A study of professional and recreational participants. Rev. Psicol. Deport..

[17] Anshel M.H., Lidor R. (2012). Talent Detection Programs in Sport: The Questionable Use of Psychological Measures. J. Sport Behav..

[18] Martin J.J., Malone L.A., Hilyer J.C. (2011). Personality and Mood in Women’s Paralympic Basketball Champions. J. Clin. Sport Psychol..

[19] Bauger L., Eisemann M., Vangberg H.C. (2014). Personality traits among junior elite athletes in Norway, and a comparison with their non-athletic counterparts. Innovative Writings in Sport and Exercise Psychology.

[20] Allen M.S., Laborde S. (2014). The Role of Personality in Sport and Physical Activity. Curr. Dir. Psychol. Sci..

[21] Eysenck H.J., Eysenck S.B.G. (2007). EPQ-A y J: Cuestionario de Personalidad Para niños y Adultos.

[22] Caprara G., Vecchione M., Barbaranelli C., Alessandri G. (2013). Emotional Stability and Affective Self-regulatory Efficacy Beliefs: Proofs of Integration between Trait Theory and Social Cognitive Theory. Eur. J. Pers..

[23] Laborde S., Guillén F., Mosley E. (2016). Positive personality-trait-like individual differences in athletes from individual- and team sports and in non-athletes. Psychol. Sport Exerc..

[24] Allen M.S., Magee C.A., Vella S.A., Laborde S. (2017). Bidirectional associations between personality and physical activity in adulthood. Heal. Psychol..

[25] Newton M., Duda J.L., Yin Z. (2000). Examination of the psychometric properties of the Perceived Motivational Climate in Sport Questionnaire-2 in a sample of female athletes. J. Sports Sci..

[26] Cecchini J., González C., Prado J., Brustad R. (2005). Relación del clima motivacional percibido con la orientación de meta, la motivación intrínseca y las opiniones y conductas de fair play. Rev. Mex. Psicol..

[27] Cervelló E., Escarti A., Balague G. (1999). Relaciones entre la orientación de metas disposicional y la satisfacción con los resultados deportivos, las creencias sobre las cusas de éxito en deporte y la diversión con la práctica deportiva. Rev. Psicol. Deport..

[28] Goudas M., Biddle S., Fox K. (1994). Perceived locus of causality, goal orientations, and perceived competence in school physical education classes. Br. J. Educ. Psychol..

[29] Murcia J.A.M., Coll D.G.-C., Garzón M.C. (2009). Preliminary Validation in Spanish of a Scale Designed to Measure Motivation in Physical Education Classes: The Perceived Locus of Causality (PLOC) Scale. Span. J. Psychol..

[30] Vallerand R.J., Rousseau F.L. (2001). Intrinsic and extrinsic motivation in sport and exercise: A review using the hierarchical model of intrinsic and extrinsic motivation. Handbook of Sport Psychology.

[31] Iglesias E., Cecchini J.A., Cueli M., González-Castro P. (2020). Análisis de la relación entre diferentes variables psicológicas en el contexto deportivo de los futbolistas. Univ. Psychol..

[32] Smith A.L., Balaguer I., Duda J.L. (2006). Goal orientation profile differences on perceived motivational climate, perceived peer relationships, and motivation-related responses of youth athletes. J. Sports Sci..

[33] Healy L.C., Ntoumanis N., Duda J.L. (2016). Goal motives and multiple-goal striving in sport and academia: A person-centered investigation of goal motives and inter-goal relations. J. Sci. Med. Sport.

[34] Martinent G., Decret J.-C., Guillet-Descas E., Isoard-Gautheur S. (2014). A reciprocal effects model of the temporal ordering of motivation and burnout among youth table tennis players in intensive training settings. J. Sports Sci..

[35] Rumpf M.C., Schneider A.S., Schneider C., Mayer H.M. (2014). Training Profiles and Motivation of Male and Female Youth Soccer Players. Int. J. Sports Sci. Coach..

[36] Wang M., Erdheim J. (2007). Does the five-factor model of personality relate to goal orientation?. Pers. Individ. Differ..

[37] Eysenck H.J., Nias D.K.B., Cox D.N. (1982). Sport and personality. Adv. Behav. Res. Ther..

[38] De Moor M.H.M., Beem A.L., Stubbe J.H., Boomsma D.I., De Geus E.J.C. (2006). Regular exercise, anxiety, depression and personality: A population-based study. Prev. Med. (Baltim).

[39] Fusté-Escolano A., Ruiz Rodríguez J. (2000). Estructura factorial de la versión reducida del “Eysenck Personality Profiler”. Psicothema.

[40] Cecchini-Estrada J.-A., Méndez-Giménez A., Cecchini C., Moulton M., Rodríguez C. (2015). Exercise and Epstein’s TARGET for treatment of depressive symptoms: A randomized study. Int. J. Clin. Health Psychol..

[41] Murcia J.A.M., Gimeno E.C., Coll D.G.-C. (2008). Relationships among Goal Orientations, Motivational Climate and Flow in Adolescent Athletes: Differences by Gender. Span. J. Psychol..

[42] Allen M.S., Greenlees I., Jones M. (2013). Personality in sport: A comprehensive review. Int. Rev. Sport Exerc. Psychol..

[43] Mckelvie S., Lemieux P., Stout D. (2003). Extraversion and Neuroticism in Contact Athletes, No Contact Athletes and Non-athletes: A Research Note. Athl. Insight Online J. Sport Psychol..

[44] Biddle S.J.H., Atkin A.J., Cavill N., Foster C. (2011). Correlates of physical activity in youth: A review of quantitative systematic reviews. Int. Rev. Sport Exerc. Psychol..

